# The General Medical Council and Voluntary Hospitals

**Published:** 1911-12-09

**Authors:** 


### THE GENERAL MEDICAL COUNCIL AND VOLUNTARY HOSPITALS.

The General Medical Council has made the following representations to the Government in regard to voluntary hospitals :—

That notwithstanding the introduction of certain amendments relating to the treatment of insured persons in voluntary hospitals and other charitable institutions, there is ground for apprehension that the national scheme established by the Bill may operate so as seriously to impair the financial stability, and therefore the efficiency, of such hospitals and institutions, and that in view of the danger thus apprehended steps should forthwith be taken by the Government to institute inquiries on this subject, and if necessary to propose legislation for the purpose of safeguarding the important interests, educational, scientific, and philanthropic, that are involved.

In his report on the Council's negotiations with the Government, Sir Donald Macalister said there was still a great danger that voluntary hospitals might suffer with regard to certain emergency cases which they would be bound to treat, and for which there was no definite provision. There was also the danger of diversion of the contributions of the workmen and employers from the hospitals to the insurance fund. It was the Council's duty to point out that that danger still remained, and to ask the Government to make an inquiry into the whole facts of the case as to how hospitals might be affected : if the inquiry proved that the Council's fears were well grounded the Government to take steps in a supplemental Bill to safeguard the interests of the voluntary hospitals. These recommendations are in precise accord with the policy which the managers of the voluntary hospitals, with the workmen's co-operation, should, in our opinion, pursue.

